# Health and Illness as Enacted Phenomena

**DOI:** 10.1007/s11245-021-09747-0

**Published:** 2021-05-19

**Authors:** Fredrik Svenaeus

**Affiliations:** Södertörn University, Huddinge, Sweden

**Keywords:** Health theory, Phenomenology of illness, Enactivism, Biopsychosocial, Existential feelings, Philosophical anthropology

## Abstract

In this paper I explore health and illness through the lens of enactivism, which is understood and developed as a bodily-based worldly-engaged phenomenology. Various health theories – biomedical, ability-based, biopsychosocial – are introduced and scrutinized from the point of view of enactivism and phenomenology. Health is ultimately argued to consist in a central world-disclosing aspect of what is called existential feelings, experienced by way of transparency and ease in carrying out important life projects. Health, in such a phenomenologically enacted understanding, is an important and in many cases necessary part of leading a good life. Illness, on the other hand, by such a phenomenological view, consist in finding oneself at mercy of unhomelike existential feelings, such as bodily pains, nausea, extreme unmotivated tiredness, depression, chronic anxiety and delusion, which make it harder and, in some cases, impossible to flourish. In illness suffering the lived body hurts, resists, or, in other ways, alienates the activities of the ill person.

## Introduction

In what follows I will investigate the significance of an enacted perspective for theories of health and illness. Enactivism is a school of thought dealing with cognition and perception from the point of view of what has recently become known as “the 4Es and an A,” namely Enacted, Embodied, Embedded, Extended and Affective (cognition and perception) (Gallagher 2017: 28). According to enactivists, an organism *enacts* its world by choosing and changing features of its environment that make sense for it in accomplishing things that matter to it. These goals may be rudimentary—to survive and reproduce—or more complex, depending upon the type of organism we are discussing.

If the organism has a brain and is conscious, or even self-conscious, the goals will be more advanced and pertain to things that are deliberated over, rather than being carried out by way of instinct and reflex. In such cases, according to enactivists, the perception and deliberation processes should be looked upon as activities in their own right in which the brain is embedded by its body and the environment, and extends to the world by way of its body when it acts. Instead of consisting in representations of the world that the brain makes of its present environment and possible futures, the reasons for acting are deliberated in the brain by way of processes in which the current state and goals of the organism are evaluatively experienced in affects which push and pull the individual to do things (Buzsáki 2019; Damasio 2018). Such reasons to act, which are evaluatively felt, bring normative issues to the analysis that extend questions about survival and reproduction of organisms to include matters of personal preferences of individuals and moral questions about what is the right thing to do.

In the case of humans, the enacted view on cognition and perception as affectively grounded, non-representative and body- and world- encompassing in nature is extended to a perspective on selfhood and personal identity that views the self as more than its brain. The self/person will by such and enacted perspective be constituted by its actions/projects in the world together with others (Noë 2009). From the enacted perspective, it is not the brain that thinks, perceives and attains a personal identity, it is the embodied person, acting in the world together with others who does these things.

What consequences will such an enacted view have for the way we think about health? Health is most often considered from the point of view of medical science, denoting a state of the (human) organism as free of diseases and therefore fit to survive and reproduce. Diseases (as well as injuries or inborn defects) are biological dysfunctions of the body studied from the *third*-person—or, rather, non-person—perspective of science. Actions performed by the embodied person are ambivalent in the sense that they can be considered from the third-person perspective of an observer, but also from the *first*-person perspective of an actor, who aims to bring something about in the world. In the paper, I will introduce theories that have tried to make use of the ability to act in the interpersonal world to conceptualize health, incorporating the first- (and second-) person perspective to supplement the naturalist third- (or, rather, non-) person focus on diseases. Such theories hold health and illness – the suffering of the ill person – to be normative concepts in contrast to the value-neutral approach of a biomedical theory.

The goal of the paper is not to show that health needs to be understood as a first-person concept instead of as a third-person concept, but that the first-person aspect will form a necessary *complement* to the third-person perspective if you embark upon the road of enactivism in this context. The theories of health that I will scrutinize are the ability-based approach of Lennart Nordenfelt (1987, 2000), the biopsychosocial model introduced by Georg Engel (1977), and, lastly, various phenomenological approaches, brought together in a sharpened version of a theory I have previously articulated and defended myself (Svenaeus 2000, 2019). The reason for focusing upon Nordenfelt’s proposal is that the concept of action is central to his health theory; the reason for focusing upon Engel’s proposal is that it has served as a framework for previous enactivist attempts to address health issues, mainly within the philosophy of psychiatry; and the reason for bringing in the phenomenologists of medicine is that they have developed the most thoroughly enacted approaches to health so far, according to the understanding of enactivism introduced above.

By way of this discussion I will also indicate how I think a phenomenology of health could make enactivism even more aware of the A (Affectivity) in the “4Es and an A,” than has so far been the case, and in what way this awareness will have significance for moral issues in medicine. From a phenomenological perspective, bodily-based moods open up the world as a space of meaning in which a person may enact possibilities as meaningful projects. Health versus illness are homelike versus alienated versions of such feelings with world-opening versus world-destroying powers, I will argue. Such a view on health and illness will have consequences for medical ethics, particularly as concerns issues of how to understand the two different perspectives of the clinical encounter: the illness sufferings presented by patients and the interpretations carried out by medical professionals. I will end the paper by briefly discussing how an enacted phenomenological view on human nature could be used to develop a slightly different approach in medical ethics than the standard principle-based one (see, further: Svenaeus 2017). Such a view will allow us to take into account the concerns of patients in a more enacted, embodied, embedded, extended and affectively enlightened way.

## Biomedical and Ability-Based Definitions of Health

The project of considering health in an enacted framework may appear far-fetched considering the way health is standardly addressed in medical science. Health, in the context of medical science, and, also, in most cases of medical practice, is focused upon only indirectly by way of the absence of disease(s) or other maladies, such as injuries, inborn defects or mental disorders. And diseases are biological phenomena rather than belonging to the person-related sphere of cognition, perception and action. From the biomedical perspective, health is not something we do, it is rather something we *have* or *do not have* depending upon the biological state or our bodies.

An influential and still standing version of the biomedical definition of (ill)health via disease was presented by Christopher Boorse in the 1970s (Boorse 1977, 1997). According to Boorse, a biological organism (human or other) is healthy if it is not afflicted by any diseases. The concept of disease is defined by Boorse a state of an organ (or organ system) which interferes with or prevents the normal functioning of the organ in question. Organs and their parts (tissues, cells) function normally, according to Boorse, when they contribute to the survival and reproduction of the organism in a statistically standard manner. Diseases may be thought of as processes rather than states – say when a virus infects an organism – but the key issue is that at some point in the invading process organ(s) of the organism reach(es) a diseased state making the bearer unhealthy (hopefully in this case only temporarily, but in some cases leading to chronic disease). Lennart Nordenfelt sums up the perspective and key points of such a biomedical health concept nicely:

Humans have a number of specific characteristics in terms of both structure and function. We have some idea of how these characteristics have contributed to the survival of individuals and the species as a whole. Through this knowledge we can picture a pattern for the life of a surviving individual. Through this vision we have laid the foundation for a biologically oriented theory of health and illness. A healthy human being, such a theory says, functions according to the pattern which is typical for the species man. A human being is unhealthy, on the other hand, if one or more of her functions deviates subnormally from this pattern (Nordenfelt 2000: 77).

There are many ways of arguing and showing that the biomedical concept of health is insufficient, at least in the case of human beings. The focus on disease is certainly not incorrect – diseases are obviously main causes of illness – but the idea that all there is to say about health could be said by way of focusing upon the functionality of the biological organism is questionable. Humans (and perhaps some other animals, too) are not only biological organisms, they are also persons who may fall ill and suffer as the consequence of other things than diseases, and who may in some cases be healthy in spite of having minor diseases if these do not bring about any sufferings.

As a matter of fact, Boorse acknowledges himself that a health theory based on medical science will always have to face the dilemmas of medical practice, including ill persons, by developing what he refers to as “disease-plus” concepts (Boorse 1997: 100). I may be currently healthy in Sweden but not in the USA, depending upon how the upper limit of a “too high” blood pressure is negotiated, and this in turn depends upon medical risk evaluations made by doctors as well as lobbying from pharmaceutical companies. However, it could be argued that health is normative in a deeper sense than being influenced by the way individual physicians, health care authorities and lobbyists define subnormal bodily functions, since persons have goals that extend, and in some cases even run counter to, their survival and reproduction (Canguilhem 1991). Humans want to achieve many more things in life than simply surviving and having offspring, and these goals are possibly important to understand the difference between health and illness.

Lennart Nordenfelt, cited above, is probably the most well-known philosopher developing such a multi-goal holistic concept of health in contrast to the biomedical version defended by Boorse and other naturalists following in his footsteps (Kingma 2014). Holistic theories consider “the human being as a whole”, the individual person, to be the healthy or not healthy in contrast to his or her biological body (Nordenfelt 1987: 12). According to Nordenfelt: “A is healthy if, and only if, A is able, given standard circumstances in his environment, to fulfill those goals which are necessary and jointly sufficient for his minimal happiness” (1987: 79); and minimal happiness is considered by Nordenfelt to be an emotional state in which the individual believes that his basic vital goals in life have been achieved (1987: 91). Nordenfelt thinks we all share some vital goals having to do with basic human needs, but he also provides space for personal variation depending upon life style preferences and the world view of each individual. One of the major reasons for being unable to reach one’s vital goals is certainly various forms of diseases afflicting the body, but such diseases do not render the individual unhealthy by themselves, but only by influencing the person’s capability to act.

Does Nordenfelt’s theory make health into an enacted concept? I would say no, since the things that make a person healthy or unhealthy are not actions in themselves but (dis)*abilities to perform* actions. Health is not an activity, and, correspondingly, illness is not a defective form of activity, they are rather things making successful actions possible or not possible. If a person is able to carry out the actions necessary to attain minimal happiness or not, is dependent not only upon the abilities of the person, but also upon the opportunities provided by the environment, a factor which Nordenfelt takes into account by specifying “standard circumstances” in his definition (1987: 79). In later works, Nordenfelt sides with the phrase “accepted circumstances,” as an alternative for “standard circumstances” (Nordenfelt 2000: 73), but the idea is still the same: to exclude situations in which the individual gets access to non-standard forms of assistance or is deprived of standard opportunities to do everyday things that matter to him in life. Thus, in Nordenfelt’s understanding, it is perfectly possible for an individual to be healthy but very unhappy – because of non-standard circumstances such as extreme poverty or war – or ill but happy – because of vital life goals being realized by fortunate circumstances involving help provided by others.

Note that also the thing that health makes possible – happiness – is not an activity in Nordenfelt’s understanding, but rather an end state of actions and other natural happenings that give rise to a belief held by the individual: that things in life are roughly the way I want them to be. The actions of a person are, so to say, the things that make a happy life possible, but happiness itself is a state of belief (Nordenfelt 2000: 86). Nordenfelt calls such a state of equilibrium, between a person’s wishes and the belief that they have been realized, an emotion, but this merely indicates that the person will standardly feel good about her situation during some periods of the time interval of being happy, not that she necessarily does so all, or even most of, the time when being happy (2000: 88). Nordenfelt clearly defends a cognitive and dispositional theory of emotion, similar to the one developed and defended by Martha Nussbaum (2001). I will return to the issue of how to consider the felt quality of emotions in presenting the main thoughts of phenomenology in relationship to enactivism below (Colombetti 2014).

Actions are defined by Nordenfelt as movements of the body carried out in attempts to reach goals aspired by the acting person more or less consciously. You may have to ask the person if the waiving of her hand is carried out in order to hit a fly or to greet you, but the action in question has to be *visible* for you in order to count as an action. It is debatable whether this is a third-person or rather a *second*-person perspective. A third-person perspective denotes a scientifically neutral study of the acting subject (turning him into an object), whereas a second-person perspective would rather make him a fellow subject in the everyday world (what phenomenologists refer to as a “life world”). In both cases, however, the acting person would be approached from outside-in rather than from inside-out (the first-person perspective of the phenomenologist). An important question, which I will return to below, is if enactivism has fully adopted the significance of the phenomenological first-person perspective in stressing the inside-out approach.

Experiences of suffering or well-being play an indirect role in Nordenfelt’s health theory, as being typically experienced in illness versus health, but they do not define health status as such, only the ability to act and the setting of vital goals do. Phenomenological theories of health and illness view things differently; from a phenomenological point of view the embodied and worldly embedded experiences of a person would be the primary focus in determining whether she is healthy or not (Svenaeus 2019). A phenomenologist will start out in the first-person perspective-manner when it comes to analyzing health and illness and doing so will have consequences for our views on what constitutes actions and activities as ways of being in the world in the first place (Aho and Aho 2008; Carel 2016).

## The Biopsychosocial Model and the Phenomenology of Health

Biomedical and ability-based definitions of health have advantages versus shortcomings, respectively. The major advantage of a biomedical naturalist understanding is that it promises a value-independent, objective way of determining if an individual is healthy or not by way of the functionality of her biological organism. The major advantage of the ability-based holistic understanding of health is that it takes into account the way persons differ in evaluating situations and setting goals for themselves in life. A phenomenological understanding of health would add to the holistic perspective that these person-based differences also concern the way individuals *experience* the state of their bodies and the environment they live in.

In order to keep the benefits of having many perspectives on health, medical researchers and practitioners have ever since Georg Engel’s seminal paper talked about “the biopsychosocial model of health” (Engel 1977). Engel’s paper, have been very influential, not least among philosophers approaching health issues from an enacted position (eg. de Haan 2020; Krueger and Colombetti 2018). It is unclear, however, how the different layers of biology, psychology, and social-cultural context relate to each other in the biopsychosocial model. Are the layers causally related or do they rather represent three different perspectives on the same thing? And, if the latter, does this mean that a person could be biologically unhealthy but still psychologically and social-culturally healthy or the other way around (or other versions of the nine options provided by three factors)? Engel seems to rely on general system-theory and talks about material and information-based flow between the levels, but it is unclear how this will solve the above mentioned problems.

My strategy in what follows will be to reinterpret the talk about biology plus psychology plus society and culture in terms of a phenomenological theory of health and explore to which extent such a view would overlap with enactivist stand-points, especially when it comes to the concepts of health and illness as such. There have been some attempts to address health issues by way of enactivism already, mainly in the field of philosophy of psychiatry (eg. de Haan 2020; Fuchs 2018; Krueger and Colombetti 2018; Seniuk 2020; Varga 2018), but I will start out with an attempt to give a *general* phenomenological characterization of health and illness (including both somatic and psychiatric illness). Referring to developed accounts, health versus illness could in a phenomenological context be understood as *homelike* versus *unhomelike* ways of finding oneself in a world (Svenaeus 2000) in which the body *dis*- versus *dys*-appears (Leder 1990) in everyday activities. Accordingly, a phenomenological approach to health will start out in the *experience* of feeling healthy or not. But as Gadamer notes in his musings on health and medical practice in the collection of essays named *The Enigma of Health*:

Without doubt it is part of our nature as living beings that our conscious self-awareness remains largely in the background so that our enjoyment of good health is constantly concealed from us. Yet despite its hidden character health none the less manifests itself in a general feeling of well-being. It shows itself above all where such a feeling of well-being means we are open to new things, ready to embark on new enterprises and, forgetful of ourselves, scarcely notice the demands and strains which are put on us. … Health is not a condition that one introspectively feels in oneself. Rather, it is a condition of being involved, of being-in-the-world, of being together with one’s fellow human beings, of active and rewarding engagement in one’s everyday tasks (Gadamer 1996, 112–113).

Health conceals itself from us, rather than standing out as a specific feeling, to the extent that it makes it possible to engage in projects opened up to us by a world shared with others. Health makes us at home with our bodies and in the world in which we are constantly striving to realize ourselves through embodied actions. Gadamer’s approach to health certainly sounds like a thoroughly enacted one, and enactivism itself, indeed, has deep phenomenological roots in relying on the philosophies developed by Heidegger (1996), Merleau-Ponty (2012) and other phenomenological thinkers (Gallagher 2017: 5). In Heidegger, the enactivists find the subject’s engaged coping with surrounding objects “being at hand” in the world, and in Merleau-Ponty they find the bodily basis of meaning-making known as “the lived body,” making possible our “being-in-the-world,” a concept coined by Heidegger and carried forward by Merleau-Ponty and others.

In contrast to health, illness does *not* conceal itself from us but rather addresses us unrelentingly. When we fall ill this condition makes itself known to us more or less immediately in a way that is hard to ignore. We feel that something is wrong by the ways our bodies show up as painful and obstructive in our everyday activities (Carel 2008; Toombs 1993). The feeling of illness is an embodied phenomenon that makes itself known by the way this embodied feeling makes it harder for us to find ourselves at home in the surrounding world when the lived body obstructs our attempted actions. As Richard Zaner puts it in his study of what he calls “the body uncanny”:

My body, like the world in which I live, has its own nature, functions, structures, and biological conditions; since it embodies me, I thus experience myself as implicated by my body and these various conditions, functions, etc. I am exposed to whatever can influence, threaten, inhibit, alter, or benefit my biological organism. Under certain conditions, it can fail me (more or less), not be capable of fulfilling my wants or desires, or even thoughts, forcing me to turn away from what I may want to do and attend to my own body: because of fatigue, hunger, thirst, disease, injury, pain, or even itches, I am forced at times to tend and attend to it, regardless, it may be, of what may well seem more urgent at the moment (Zaner 1981: 52).

The lived body, which turns alien in illness, is structurally linked to meaning layers of everyday actions as well as to existential thoughts and projects by way of what Heidegger calls attunement (Heidegger 1996). The unhomelike feeling of illness will protrude from the lived body to attune everyday activities and, sometimes also, existential projects articulated and carried out by way of cultural meaning-patterns (Svenaeus 2017: chapter 3). The phenomenologist can make sense of the three different zones of biology, psychology and society-culture as belonging to the same lived realm, forming layers of meaning enacted by human beings. This connectedness is what remains unexplained in standard talk about a bio-psycho-social model of health (de Haan 2020).

By allotting the biological functions of the body a basic role in meaning making, the phenomenologist can also provide a place for diseases and other maladies in health theory. In the same way that our bodily disposition and the physiology of the human brain – connected to the rest of the body – are vital to understand a person’s way of being in the world (Gallagher 2005), the dysfunctions affecting our bodily physiology are linked to (failed) attempts of sense making (Svenaeus 2019). Diseases *typically* lead to unhomelike being in the world; and even if they do not *necessarily* do so, the way they make it harder to accommodate personal life projects and make us feel at unease (compare “dis-ease”) with our bodies reminds us of the basic causal relationship between nature and culture (Canguilhem 1991). Reversibly, the ways in which stress or sadness have negative effects on health and, in the long run, are linked to development of mental disorders and somatic diseases, bear witness of a causal effect issued by culture on nature (Leder 2016).

## Phenomenology and the Concepts of Action and Feeling

The most basic phenomenological concept is arguably *intentionality*, experiences had by a subject is *about* something, *directed towards* things in the world. So far I have not made use of the terminology of intentionality, but instead relied on the parallel expression of “being-in-the-world” (with or without hyphens). This reflects a preference for a more enacted phenomenology (Heidegger rather than Husserl) in which understanding actions of a self is chosen as the conceptual framework, instead of perceiving acts of consciousness, when a subject makes sense of things in the world. Nevertheless, Husserl’s (and Merleau-Ponty’s) terminology of intentionality could serve us well in showing how the phenomenological view on action is different from the third-person perspective we find in analytical action-based philosophy, such as the health theory developed by Nordenfelt that we explored above. Recall that action in such a theory always refers to movements of the human body that are visible from a third-person (or second-person) perspective. For the phenomenologist, starting out within the framework of intentional acts, action will turn out to be a more inclusive concept, involving more than witnessed movements of the body.

To start with, not only moving one’s limbs and using one’s vocal apparatus will count as actions for the phenomenologist, but also, for instance, the mere *looking* at something will be a paramount example of a meaningful act and action, since the person by way of looking fixes his gaze on something in the environment. Nordenfelt could counter that such looking at things is surely visible for a by-standing party by way of perceiving the ways the eyes, and perhaps the whole attentive lived body, of the other person are turned to certain objects. You will clearly notice if another person looks at *you*, to take the most obvious example. Perhaps you will also be able to perceive if the other person is listening to something or someone, or smelling something, but will you be able to see if she is attentively sensing the uncomfortable feel of her tight sitting bra or her high heel shoes? Perhaps, in some situations, if it shows by way of her body language and/or if you have had similar experiences making you extra sensitive to the cues.

There is one thing, however, that, under most circumstances, you will have no chance of perceiving, at least in any detail or with any sense of certitude, and that is the *thoughts* of the other person. Yet, those thoughts are surely intentional in the phenomenological sense of being about something, and they could be considered as, at least imaginary, actions (Buzsáki 2019; Gallagher 2017). Heidegger’s major concept for describing our ways of being in the world in taking care of things is *understanding*, and this term covers everything from striking nails with the hammer to imagining how the house will look when it is ready and the mathematical operations carried out in order to get the proportions right in preparing the timber for building it (Heidegger 1996).

What will such an expanded conceptual scope of action mean for health theory? If health is considered to be a homelike being in the world and illness is looked upon as a breakdown of ongoing transparent activity, it could be argued that health versus illness are *enacted* in a deep sense. They are not only related to action indirectly in making us able or disabled to act – such as is the case in a naturalist theory of health as absence of diseases – they are in themselves different forms of activity patterns. Many phenomenologists of health have made use of Heidegger’s example of the broken hammer as indirectly making us aware of the normal manner of handling the “ready to hand” things in the world, and compared it to cases in which “body tools” break down, that is: cases of illness (Carel 2016: 60–63; Leder 1990: 83–84; Svenaeus 2000: 108–109; Toombs 1993: 136 ff.). This parallel between break downs of outer tools and body tools occurring in the activities that constitute our being in the world, comes close to considering illness and health as activity patterns in which things and projects appear as meaningful for a person. The scenario could be expanded to cover psychiatry, although it is the brain rather than the hand or the kidney that breaks down in cases of mental illness. Sanneke de Haan, for instance, in “An enacted approach to psychiatry” claims psychiatric disorders to be “disorders of sense making” in which “the evaluative interactions of a person and her world go astray” (2020: 10).

The problem with such an enacted view on health and illness as de Haan’s, I would argue, is that it tends to conflate the meaning-making activities themselves with that which makes them possible and provides them with a conceptual space to materialize in: world-opening feelings. The order of priority between health versus illness and meaningful versus meaning-lacking activity is not temporal but rather logical, since certain feelings, namely the ones that Heidegger names attunements or moods, and which Matthew Ratcliffe has recently rechristened as “existential feelings” (2008, 2015), make being in the world possible in the first place. Being in the world is always attuned in some way or the other. Bodily-based moods open up the world as a space of meaning in which to enact possibilities as meaningful projects. Health versus illness are homelike versus alienated versions of such feelings with world-opening versus world-destroying powers, I would argue.

Existential feelings originate and are felt by way of the lived body, but they are also meaningful in the sense of providing patterns of understanding for things mattering to us in the world, patterns that they create and encircle. Health is not one such particular feeling, it is rather the world-opening potential that all existential feelings harbor, making them transparent and allowing us to direct our attention beyond the lived body and the mood itself to the things, persons and projects that show up as mattering to us in the world through the feelings. Illness is not one but rather many different existential feelings in which the world-opening potential in various ways threatens to break down: bodily pains, nausea, extreme unmotivated tiredness, depression, chronic anxiety and delusion, to mention some examples (which could be further divided and split up by, for instance, specifying different types of bodily pains and anxieties). Some enactivists have acknowledged the role of feelings in making sense of everyday activities and in the face-to-face encounter between persons (Colombetti 2013: chapter 7; Gallagher 2017: chapter 8; DiPaolo et al. 2018: part III), but enactivists have not so far developed the role of affectivity in health theory.

## Health, Flourishing and Suffering

In the ability-based model we saw how health and happiness were related by way of actions. According to Nordenfelt, health is the ability to realize the vital goals constituting minimal happiness and this is done by way of acting. Happiness is not itself a form of activity but rather a set of emotional beliefs held by the subject. Nussbaum, who defends a similar theory of emotion as the one found in Nordenfelt’s work, would add that happiness is not merely a set of beliefs but also a set of *capabilities*, involving abilities and *opportunities* to do things that are important for the person’s well-being (Nussbaum 2011). From a phenomenological point of view, indebted to Aristotle, happiness could be considered as an activity pattern in which the person realizes his aims and potentials together with others in the world, a way of *flourishing* (Guignon 2004; Madison 2013). We are reminded of the characterization of health offered by Gadamer, quoted above, except that the well-being of flourishing would not only refer to an openness for realizing new things in the world, but also to having *accomplished* at least parts of what one aspires to in life and feeling that this matters.

Suffering, in contrast, would refer to situations in which a person is prevented from flourishing and experiences this as a loss in her being in the world, making her less of herself, so to say, in my study *Phenomenological Bioethics: Medical Technologies, Human Suffering, and the Meaning of Being Alive* I put it like this:

Suffering is an alienating mood overcoming a person and engaging her in a struggle to remain at home in the face of the loss of meaning and purpose in life. Such a mood (or combination of moods) involve painful experiences at different levels that are connected but are nevertheless distinguishable by being primarily about, firstly, my embodiment, secondly, my engagement in the world together with others, and, thirdly, my core life-narrative values (Svenaeus 2017: 36).

Illness suffering is primarily a bodily suffering, but such bodily illness-moods are enacted in the world and ultimately influence the cultural-existential level also (core life-narrative values to be realized), when a person is prevented from flourishing due to somatic illness. In psychiatric illness, the role of the lived body is less explicit, since the illness mood is not always perceptible by way of certain parts of the body standing out as painful and disturbing (Leder 1990). However, it could nevertheless be argued in such cases that the being in the world of the person has turned unhomelike in a bodily manner, since psychiatric illness-moods typically affect the supporting role of lived-bodily intentionality. Matthew Ratcliffe shows in his studies on psychosis, depression and anxiety disorders how the whole-body experience will change in existential feelings typical for psychiatric illness, making the body feel irreal, heavy or panicky in a way that obstructs everyday activities and flourishing with others (Ratcliffe 2008, 2015) and many other phenomenologists in the field of psychiatry have made similar observations (Fuchs 2000).

Health supports human flourishing just as illness tends to obstruct such flourishing. Flourishing in the world by way of realizing one’s potentialities and aims together with others could in many cases be possible despite illness, but the alienating force of illness moods make it much harder, and sometimes even impossible, to flourish. Health, on the other hand, constitutes a basic disclosive and supportive power by way of the world-opening potential at hand in healthy existential feelings, but since being in the world could turn unhomelike for other reasons than illness, health does not guarantee flourishing (Svenaeus 2017: chapter 5). Persons suffer for many other reasons than falling ill and such forms of ill-fated flourishing could be just as painful despite the reason and character of the suffering being non-bodily. Think of losing one’s job and being deprived of social contacts as a result of the lock-downs carried out to stop the spread of the corona virus during 2020. Or losing a loved one who died as a result of being infected by the virus. In all these cases a person will experience suffering for other reasons than illness, in contrast to the loved one, who clearly experienced illness-suffering during the last weeks of her life.

Despite not being an activity in its own right, but rather a supportive experience, health is dynamically related to the activity patterns of human flourishing taking place in the meaningful world shared with others. Illness and other forms of human suffering are moods/existential feelings in and by which such flourishing efforts are stranded. Certain forms of suffering, in and by which we are brought to a deeper understanding of our human predicament, could in the long run be important to cultivate individually enlightened flourishing (Aho and Aho 2008). We often call such forms of human suffering *existential suffering*, at least when the suffering turns out to have gained us retrospectively. When it does not, we tend to give it a medical name, such as “major depressive disorder,” or some of the other about 300 psychiatric diagnoses to be found in listing manuals, such as the DSM-5 (American Psychiatric Organization 2013). This frequent wavering between existential insights versus psychiatric illness indicates a more complex relationship between the zones of biology, psychology and social-cultural context in the case of psychiatry, compared to somatic medicine, not forgetting that the latent dualism in distinguishing layers of meaning making should be avoided (Aho 2018).

## Health, Philosophical Anthropology and Medical Ethics

Even though health versus illness are not activities in themselves, but rather world-opening versus world-closing powers experienced by way of existential feelings (or bodily attunements/moods if we use Heidegger’s terminology, rather than Ratcliffe’s), they are conceptually related to human meaning-making activities. A person’s health versus illness makes way for a homelike versus unhomelike being in the world in which the body dis- versus dys-appears and this means that the phenomenon of health belongs to philosophical anthropology (Leder 1990; Svenaeus 2000; Zaner 1981). Health and illness are normative concepts because they define human experiences that are good versus bad in nature.

Importantly, feeling healthy is not an experience of well-being in the sense of feeling happy and prosperous. Health is rather a non-apparent attunement that makes it possible to flourish and experience well-being in the stronger sense of a positive mood. Illness, in contrast, is an unpleasant existential feeling making itself known by way of the body dys-appearing instead of dis-appearing in everyday activities. By way of illness moods not only one’s body but also the surrounding world appear as alien in nature (Svenaeus 2019). In the case of psychiatric illness, the dys-appearance takes on a more existential character by relating to patters of meaning making that are rather cognitive than practical in style (Aho 2018). Nevertheless, the unhomelike experiences suffered by psychiatric patients are also bodily and perceptual, linked to possibilities for action in the deeper and expanded sense that enactivism suggests (Gallagher 2017).

Another possible counter argument to the idea that health consists in a homelike being in the world is the example of pregnancy. Is this not an obvious example of experiencing one’s own bodily being as alien and unhomelike in the sense that another living creature is developing in the womb? And yet being pregnant is clearly not an example of being ill. Iris Marion Young argues that the experiences of pregnancy, including the event of quickening when the pregnant woman feels the presence of the fetus kicking in her belly, are not alienating in themselves (Young 2005). What alienates the life of pregnant and birthing women, according to Young, is the medical-technological gaze associated with the equipment of maternal care.

I think Young and other feminist scholars are right in pointing towards the risks of unnecessarily medicalizing pregnancy, and also in claiming that pregnancy, despite involving the experience of “an alien,” is most often not an alienating experience in this regard. There is a clear difference between, for instance, the typical occurrence of morning sickness in early pregnancy and the events of quickening. The difference is between the experiences of the lived body as alien – in this case, in nausea – and the experiences of another living being in my body who is not an alien. The fetus may to some extent be perceived as an unwelcome stranger – particularly if the pregnancy is unwanted – but in most cases, quickening is referred to as the first *contact* with the baby to come. To feel the fetus is to feel the *togetherness* of mother and child, and this feeling is generally not referred to by the pregnant woman as alienating, but as the feeling of a different, and in some ways, *fuller* state of being (Bornemark 2015).

The badness of illness suffering concerns the being in the world as such, which is reduced and threatened when a feeling of alienness reverberates in the very process of meaning making. Illness is not a suffering that is necessarily *about* certain things in life, or, even less, about a philosophical search for ultimate life meaning. Nevertheless, illness suffering may expand to cover such issues when important life projects are shattered or the ill person is no longer able to persist in and by way of her pre-illness identity (Svenaeus 2017: chapter 2). The badness of illness suffering is *one* form of human badness possibly, but not necessarily, linked to other forms of badness, such as political suffering or suffering that is the result of having bad luck in life (in other ways than falling ill by way of diseases).

Human persons lead their lives by way of suffering and flourishing processes. Since illness and health form parts of human suffering and flourishing, these concepts apply to *persons* and not to biological bodies (including brains). Note that the biomedical and ability-based health concepts would not find a space for illness and health within the realm of philosophical anthropology in this sense. Diseases afflict biological, not lived, bodies and abilities to act are dependent upon the physical state of human bodies, not the way these bodies are experienced as world-disclosing. Biological dysfunctions and physical abilities are clearly *causally* related to illness and health in the phenomenologically enacted sense, but they are not states of illness or health in themselves, since they are not *enacted experiences* of a person. This is the main difference between third-person and first-person health theory.

Philosophical anthropology is a term which is often used to talk about two different things that are closely related. First, it can refer to the attempt to develop an ontology of human being on philosophical ground (Hartung 2018). In this sense there has been philosophical anthropology projects going on at least since the time when Plato wrote his dialogues. Most important philosophers in the Western tradition – such as Rousseau, Kant or Nietzsche – have developed or adopted an, at least, implicitly, such a philosophical anthropology. Second, the term could refer to a specific philosophical tradition in German twentieth-century philosophy, starting out with Max Scheler and Helmuth Plessner and including names such as Erwin Straus, Frederik Buytendijk, Jan Hendrik van den Berg and Arnold Gehlen (Schlossberger 2019). These philosophers were aligned with the phenomenological tradition but in addition to the phenomenological mind-set they nurtured an interest for medicine and biology – some of them were physicians – and they wanted to combine phenomenology with empirical approaches in their theories about human being.

Health issues formed part of the theories of some philosophical anthropologists – Buytendijk and van den Berg are good examples – but health and illness were rarely focused upon explicitly. Rather than developing a phenomenology of health and illness, the philosophical anthropologists tried to show how knowledge about biological functions and diseases in medicine must be complemented by knowledge about human beings as conscious and world-related creatures. This was especially so in matters pertaining to medical ethics, in which the philosophical anthropologists aimed to show that the patient must be approached as a person rather than a set of biological dysfunctions, only (Gadamer 1996).

By moving health and illness explicitly into the field of philosophical anthropology we can see how the health and illness concepts become meaningful for medical ethics. The enacted phenomenological approach to health and illness which I have developed above show why human beings must be approached in health care as embodied, worldly embedded, attuned and flourishing creatures, instead of merely rational agents to be informed about their medical condition (Svenaeus 2017: chapter 1). Enhancing and respecting autonomy will expand to cover issues having to do with *authenticity* if we complement disease process with illness suffering as phenomena to take into consideration in health care (Aho 2018). This represents an enacted development, since the focus will be upon the world of the patient in which things attain meaning for reasons of everyday and personalized activities (in the broad sense of flourishing), which have become threatened by a medical condition.

To understand the patient’s feelings, everyday life and self-understanding, which is necessary in dealing with psychiatric illness and severe and/or chronic somatic illness, the physician, nurse or other health-care professional needs to develop and have *empathy* for the patient. However, empathy is not some kind of humanistic icing on the medical cake, a way of feeling sorry for or showing that one cares for the patient. It is rather the main gate and high road to develop knowledge about the patient as a suffering human being in and through a dialogue with him (Svenaeus 2017: chapter 4). Such professional knowledge about illness is necessary to complement the knowledge about diseases in forming a clinical judgement about what is to be *done* when a diagnosis has been established. In cases that are challenging from an explicit ethical standpoint, for instance, when a patient asks for assisted dying, or, when a patient refuses treatment even though this will be harmful for him, knowledge about individualized suffering and collapsed flourishing is even more crucial to reach good (or less bad) decisions in health-care. The threads of thoughts forming the movement and program of enactivism could support such efforts, particularly by approaching health and illness as enacted concept, even though they do not equal actions or activities in themselves. By acknowledging the role played by existential feelings (embodied moods) in human being in the world, enactivists could adopt a phenomenological perspective on health and illness and make contributions to the field of medical ethics in a more substantial way.

## Conclusion

I have explored the concept of health through the lens of enactivism, understood as a bodily-based, worldly-engaged phenomenology. From the point of view of enacted phenomenology, health is not only causally but also conceptually related to the experiences and activities of a human person. If health is an existential feeling (bodily-based attunement/mood), or, rather, a central world-disclosing aspect of various existential feelings, then health is an important and in many cases necessary part of leading a good life. Illness, on the other hand, by such a phenomenological view consist in finding oneself at mercy of unhomelike existential feelings, which make it harder and, in some cases, impossible to flourish. In illness suffering the lived body hurts, resists, or, in other ways, alienates the being in the world of the ill person.

Health and illness are normative concepts, not only because health is good and illness is bad for a person, but also because this goodness, and, especially, badness is *felt* by the flourishing versus suffering person. The existential feelings of health versus illness is the reason why our medical condition ultimately *matters* to us and why we seek out health care professionals to get help. In medical ethics, health and illness should be viewed from the point of view of a philosophical anthropology that incorporates and spells out the normative aspect of health and illness by linking them to flourishing and suffering in the life of a patient. The moral dilemmas dealt with in medical ethics will gain from being explored by way of an enacted and bodily-attuned perspective, focusing upon the being in the world of all involved parties.
